# *Notes From the Field:* Coccidioidomycosis Outbreak Among Wildland Firefighters — California, 2021

**DOI:** 10.15585/mmwr.mm7134a4

**Published:** 2022-08-26

**Authors:** Marisa A.P. Donnelly, Dorothy Maffei, Gail L. Sondermeyer Cooksey, Thomas J. Ferguson, Seema Jain, Duc Vugia, Barbara L. Materna, Amanda Kamali

**Affiliations:** ^1^California Department of Public Health; ^2^Epidemic Intelligence Service, CDC; ^3^Council of State and Territorial Epidemiologists, Atlanta, Georgia; ^4^California Department of Forestry and Fire Protection; ^5^University of California Davis School of Medicine, Sacramento, California.

Coccidioidomycosis, also known as Valley fever, is caused by inhalation of spores of the soil-dwelling fungi *Coccidioides* spp. Although most illness is mild, coccidioidomycosis can cause severe disease resulting in hospitalization or death. On July 28, 2021, the California Department of Forestry and Fire Protection (CAL FIRE) notified the California Department of Public Health (CDPH) of seven wildland firefighters from two crews who had respiratory illness. Crew A (19 members) and crew B (21 members) had worked on wildfires in late June 2021 near the Tehachapi Mountains, a California region with historically high coccidioidomycosis incidence.* Among the seven symptomatic firefighters, three cases of coccidioidomycosis were laboratory-confirmed; two patients developed severe disease. All three firefighters with confirmed coccidioidomycosis reported working in dusty conditions without wearing respiratory protection. Because no vaccine for coccidioidomycosis currently exists, correct use of respiratory protection is important for preventing coccidioidomycosis, especially in regions with high disease incidence.

During July 17–August 4, 2021, the seven ill firefighters each visited an emergency department two or three times with cough, chest pain, or shortness of breath; all received negative test results for SARS-CoV-2, the virus that causes COVID-19. Three of the seven firefighters were hospitalized, had serologic test results that were positive for coccidioidomycosis, and were treated with antifungal medication. CDPH interviewed these three patients and reviewed their medical records. Coccidioidomycosis serologic test results for the other four firefighters were negative; however, repeat serology is often suggested if coccidioidomycosis is suspected.^†^ Two of these four were retested and results remained negative and were managed in ambulatory clinics, and two were lost to follow-up. All confirmed cases occurred in patients who worked on crew B, resulting in an attack rate for confirmed cases of 14.3% (three of 21).

The three confirmed cases occurred in men aged 25–34 years, none of whom had any remarkable past medical history. Two patients reported Hispanic or Latino race or ethnicity, and one did not report race or ethnicity. Length of hospital stay ranged from 8 to 17 days. All three patients were treated with the antifungal fluconazole; interval from illness onset to commencement of treatment ranged from 10 to 12 days.

Illness onset and work history dates suggested that *Coccidioides* exposure likely occurred during a 3-day fire near the Tehachapi Mountains. All three patients reported digging trenches and “mopping up” the fire, which included digging and moving soil, with heavy dust exposure and without respiratory protection. All had been fit-tested for a respirator and reported having been trained to minimize dust exposure.

Coccidioidomycosis outbreaks have been reported among wildland firefighters in California, where job-related soil and dust exposure in areas with coccidioidomycosis increases the risk for infection (*1*,*2*). The fungus that causes coccidioidomycosis is endemic in the soil in the southwestern United States, particularly Arizona and California, and in parts of Mexico and Central and South America^§^; endemicity is also likely expanding (*3*). Use of respiratory protection is challenging in wildland firefighting because of concerns about respirator flammability and compatibility with other equipment, as well as the hot, strenuous nature of the emergency-related work. Despite these challenges, fire agencies could consider evaluating the feasibility of respirator use under specific conditions (e.g., during dust-generating activities away from active burning) and adopt policies accordingly.

Early recognition of coccidioidomycosis and disease management are essential to mitigating severity (*4*). CDPH has previously recommended that all California wildland firefighters receive coccidioidomycosis training regarding exposure risks, prevention, and when to seek care^¶^ (*2*); CAL FIRE policy is to conduct this training at the beginning of each fire season. Based on findings of this investigation, CDPH recommends safety briefings on coccidioidomycosis prevention, such as use of respirator protection or wetting of soil before disturbance, before deployment to, and return from, possible areas with endemic *Coccidioides* spp. During this outbreak, CAL FIRE was proactive in recommending coccidioidomycosis testing; cases were diagnosed within 12 days, compared with a median of 55 days from illness onset to diagnosis reported in an Arizona study (*5*). California health care providers should ask patients with respiratory illness about work location, high-risk occupations, and exposure to soil disturbance. Providers should also consider that signs and symptoms of coccidioidomycosis might be similar to those of COVID-19 to avoid unnecessary delays in diagnosis. As frequency of coccidioidomycosis and wildfires increase in California, exploration of protective equipment and additional training are needed to better protect wildland firefighters (*3*).

## Footnotes

*
https://www.cdph.ca.gov/Programs/CID/DCDC/CDPH%20Document%20Library/CocciEpiSummary2018.pdf

†
https://vfce.arizona.edu/valley-fever-people/order-right-tests

§
https://www.cdc.gov/fungal/diseases/coccidioidomycosis/maps.html

¶
https://www.cdph.ca.gov/Programs/CCDPHP/DEODC/OHB/Pages/Cocci.aspx
